# Renal structural image processing techniques: a systematic review

**DOI:** 10.1080/0886022X.2019.1572016

**Published:** 2019-02-12

**Authors:** Shiva Asadzadeh, Hamid Tayebi Khosroshahi, Behzad Abedi, Yaghoob Ghasemi, Saeed Meshgini

**Affiliations:** a Department of Electrical and Computer Engineering, Tabriz University, Tabriz, Iran;; b Department of Internal Medicine, Tabriz University of Medical Sciences, Tabriz, Iran;; c Medical Bioengineering Department, School of Advanced Medical Sciences, Tabriz University of Medical Sciences, Tabriz, Iran;; d Department of Medical Biotechnology, Faculty of Advanced Medical Sciences, Tabriz University of Medical Sciences, Tabriz, Iran;; e Department of Electrical Engineering, University of Tabriz, Tabriz, Iran

**Keywords:** Biopsy, Glomeruli segmentation, image analysis, pathology, renal

## Abstract

**Background and objective:** Renal disease, such as nephritis and nephropathy, is very harmful to human health. Accordingly, how to achieve early diagnosis and enhance treatment for kidney disorders would be the important lesion. Nevertheless, the clues from the clinical data, such as biochemistry examination, serological examination, and radiological studies are quite indirect and limited. It is no doubt that pathological examination of kidney will supply the direct evidence. There is a requirement for greater understanding of image processing techniques for renal diagnosis to optimize treatment and patient care.

**Methods:** This study aims to systematically review the literature on publications that has been used image processing methods on pathological microscopic image for renal diagnosis.

**Results:** Nine included studies revealed image analysis techniques for the diagnosis of renal abnormalities on pathological microscopic image, renal image studies are clustered as follows: Glomeruli Segmentation and analysis of the Glomerular basement membrane (55/55%), Blood vessels and tubules classification and detection (22/22%) and The Grading of renal cell carcinomas (22/22%).

**Conclusions:** A medical image analysis method should provide an auto-adaptive and no external-human action dependency. In addition, since medical systems should have special characteristics such as high accuracy and reliability then clinical validation is highly recommended. New high-quality studies based on Moore neighborhood contour tracking method for glomeruli segmentation and using powerful texture analysis techniques such as the local binary pattern are recommended.

## 1. Introduction

The kidney is an organ with complex organogenesis, predisposing to many congenital and acquired type of anomaly during development and exposed to some type of hematogenous and lower urinary tract origin insults. Each nephron, as a functional unit of kidney, includes the renal corpuscle and tubules. The renal corpuscle contains Bowman’s capsule and the glomerulus [1,2]. The filtration barriers on the glomerulus comprise fenestrated endothelial cells, the glomerular basement membrane (GBM), and the visceral epithelium (podocytes) that prevent passage of negatively charged molecules such as albumin (about 3.6 nm) through the barrier [3].

The basement membrane of glomeruli has an important role in structural support and functional operation of the glomerulus that mainly composed of laminin and collagen IV, and contains three layers: the lamina rara interna, the lamina densa, and the lamina rara externa. The GBM width is nearly twice the thickness of other most of human basement membranes, with the range of 300 to 350 nm [1,2].

A renal biopsy is a procedure used to extract kidney tissue for laboratory analysis. At the present time, imaging guided renal biopsy is used to provide diagnoses in most types of primary and secondary renal diseases. It has been claimed that renal biopsy can provide a link between diagnosis of renal disease and its pathological conditions. Pathological examination of biopsy specimen is time consuming and can take several days to complete and need expert renal pathologist [4].

The renal biopsy contains not only glomerulus but also other tissues. The kidney tissue has a lot of blood vessels and tubules. It is important for doctors to separate these vessels and tubules from each other and determine their health in most kidney transplants.

Accordingly, how to achieve early diagnosis and enhance treatment for kidney disorders would be the important lesion. Nevertheless, the clues from the clinical data, such as biochemistry examination, serological examination and radiological studies are quite indirect and limited. From the literatures [5], it is no doubt that pathological examination of kidney will supply the direct evidence and be the most important for treatment decision making. In kidney disease studies, tissue image will be useful in detecting any abnormality. These images are CT scans, MRI, ultrasound, and histological images. Computer-aided diagnoses have been applied to several methods of diagnostic imaging [6], all of which are generally based on radiological images. Within a few years, this field of research will be also expanding in other medical fields, such as histopathology [7], where images are completely different from radiological ones.

In the recent years, several different practical techniques have been proposed to improve the efficiency of renal biopsy by using different guiding techniques. There is a requirement for greater understanding of image processing techniques for renal diagnosis to optimize treatment and patient care. The objective of this systematic review was to identify publications that have been used image processing methods on pathological microscopic image for renal diagnosis. It was envisaged that this review would aid clinical practice by informing future research and the development to diagnosis and treatment technique in renal patients.

## 2. Methods

### 2.1. Research questions

Considering the above topics, this article focuses on the following two research questions:

- (RQ1) which image processing techniques have been used to date to distinguish renal abnormality?
- (RQ2) What are the implications for future research on diagnosis and treatment techniques in renal patients?

### 2.2. Article selection criteria

The review included studies that focused on the following criteria: (1) presented an image processing method to detect renal component and their abnormality, (2) used renal histopathology images, (3) *demonstrated* numerical and perspicuous results, and (4) were written in English. Age and disease were not as limiting factor of studies.

### 2.3. Search strategy

The principles in the Preferred Reporting Items for Systematic Reviews and Meta-Analyses (PRISMA) statement were used in this review [8]. To determine the seminal works related to image processing techniques on liver diagnosis, a review of the literature was undertaken through a search of following databases: PUBMED Digital Library, IEEE Digital Library, and Science Direct. Only the studies published from the year 2010 until 14 June 2018. This study focused on the inclusion criteria meeting in the literature, was evaluated by two independent researchers, and the agreement of both the parties determined studies suitability.

### 2.4. Statistical analysis

Table 1 shows metrics to evaluate the performance of the studies.

**Table 1.** Included studies.

Publication(s)	Year	Objectives	Study details	Methodology	Results and conclusions
CH. MAAYAN [9]	1979	Renal tubules detection	The image was projected onto an EMR image dissector (Model658A, Schlumberger) digitized and fed into a PDP-15/76 computer. The dissector consists of a sensor in which the optical image is transformed.	Thresholding	-----
Christine François [10]	1997	The Grading of renal cell carcinomas	The present series covers 65 cases of RCC from the Jules Bordet Institute and the Erasmus Hospital in Brussels. All the patients, 37 men and 28 women, underwent simple or radical nephrectomy between 1990 and 1995. The patients 'ages ranged from 27 to 85 years, with a mean of 59 years.	The decision tree and production rule methods	Accuracy:76%
Hyun-Ju Choi [11]	2007	Grading of renal cell carcinoma	Eight cases of RCC. The tissues were cut into 20-_m sections, stained with propidium iodide (PI) containing RNase A (final concentration, 0.5 mg/mL). There were a total of 50 slices for each volume data, and each slice was a 24-bit/pixel image with a size of 512 × 512 pixels.	3D morphological analysis	Accuracy:77%
Ilya Kamenetsky [12]	2008	Segmentation and analysis of the Glomerular basement membrane	The images were acquired using a Hitachi H-7000 TEM system with magnification in the range of 1,500 to 17,000. The dataset used includes a total of 34 TEM images of renal biopsy samples of six patients: one patient with abnormally thin GBM, one with abnormally variable GBM width, two with normal GBM, and two with abnormally thick GBM.	The split and merge method	Average and standard deviation of the errors: 12*±* 9
Jun Zhang [13]	2008	Glomeruli Segmentation	Normal glomerular basement membranes images are used.	A kind of feature operator based on the definition of the cavum boundary is proposed in this article. According to this operator, a nonlinear thresholding surface can be constructed by neural network, and the appropriate surface can be selected to enhance the cavum boundary by the fault tolerance analysis.	The minimum error is decreasing with the increasing of training time. The minimum error is unchangeable after 1000000 times training and the value equals to 5.420481.
Hai-Shan Wu [14]	2010	Measurement of basement membrane thickness	The studies detailed below regarding normal glomerular basement membranes utilized a core needle biopsy from a 63-year-old man who had a liver transplant 4 years prior to the biopsy and recently developed proteinuria.	The gaps between successive input points are lineally interpolated. A nonlinear mapping is applied to straighten the curved central line. Two distance functions of edges to the central line are constructed. The smooth envelope lines are obtained by repetitive applications of a linear low-pass filtering followed by a comparing and selecting process. The boundaries of the glomerular basement membrane are obtained from the inverse mapping of the envelope functions.	Average metric GBM thickness in 1:63-year-old man: 0.37098μm or 371 nm. 2: 39-year-old woman: 193.6 nm
Ravi M [15]	2015	Detection of Glomerulosclerosis in Diabetic Nephropathy	The set of two database of 50 glomerulus images from a type 1 diabetic (T1DM) patient with diffuse and nodular mesangial expansion and afferent arteriolar have been used.	Glomerulus segmentation with active contours based on Chan-Vese algorithm	Accuracy (%):86
Vito Antonio Bevilacqua [16]	2016	Blood vessels and tubules classification	Materials consist of 10 Kidney Biopsy Slides (KBS) with periodic acid Schiff (PAS) staining. The regions of interest (ROI) identified by expert are in total 221:71 vessels and 150 tubules.KBS preparation and digital acquisition have been conducted by expert technicians at the Department of Emergency and Organ Transplantation (DETO) of the University of Bari Aldo Moro (Italy). Each slice is a red green blue (RGB) format image with a resolution of 0.50 μm/pixel.	The supervised Artificial Neural Networks (ANNs) architecture based on error back propagation training algorithm based on Haralick features	Accuracy:93% Precision :91% Recall :91%
Taras Kotyk [17]	2016	Measurement of glomerulus diameter and Bowman’s space width	The experimental work was performed using a dataset of renal rat images obtained in the Anatomy Dept. Laboratory, Faculty of Medicine, Tanta University, Egypt. The tested specimen images were acquired using a light microscope with a magnification of 1000×). In this current experimental study, selected albino rats’ samples of aged 2 months (regardless their gender) are captured from a pool of 2–6 months aged rats. The dataset was a mix of both control and experimental affected groups (6 and 15 elements, respectively.	The particles analyzer technique based on median filter for morphological image processing to detect the renal corpuscle objects	Glomerulus’s diameter in the affected group: 177.03 ± 54.48 μm the control group 97.40 ± 19.02 μm. Bowman’s space width 6.18 ± 1.49 μm for the control group, and 12.04 ± 3.13 μm for the affected group.

## 3. Results

**Figure 1.**
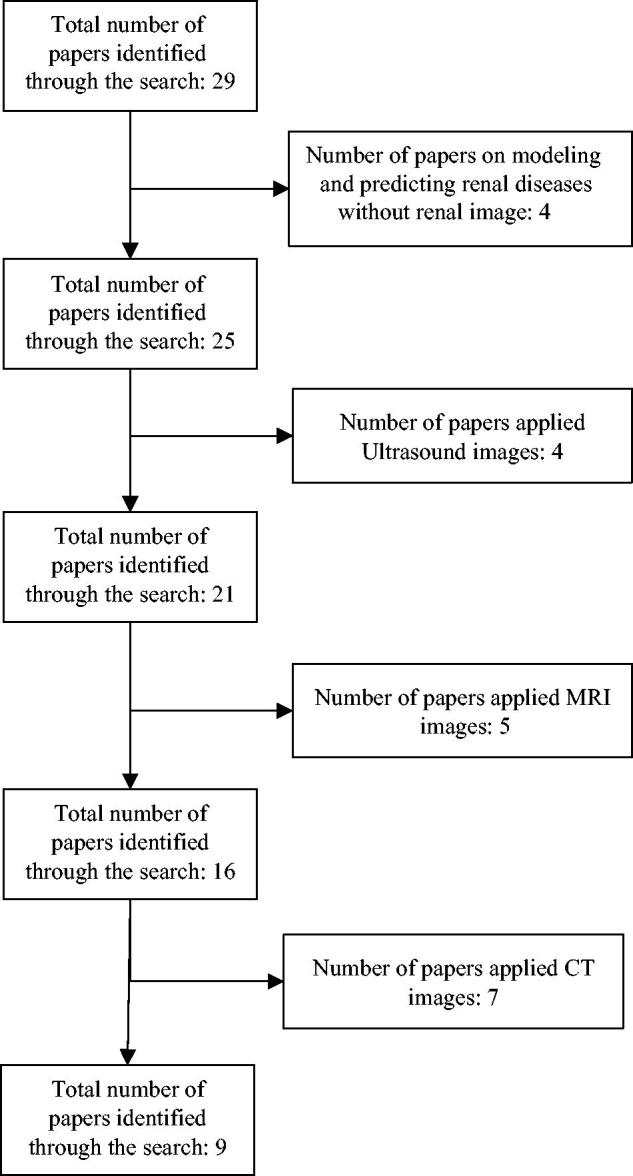
PRISMA diagram of the systematic review. PRISMA, Preferred Reporting Items for Systematic Reviews and Meta-Analyses.

Figure 1 shows the selection process for articles included in the systematic review. A total of 31 articles were identified in the original literature search. In this article, we are looking for researches in the field of pathologic image processing. In the collected studies, four cases of articles were about modeling and predicting renal diseases without renal image and did not match the defined criteria. Also, we did not consider 16 articles that used Ultrasound, CT, and MRI images in these studies for this systematic review. In summary, our review examined nine papers.

**Figure 2.**
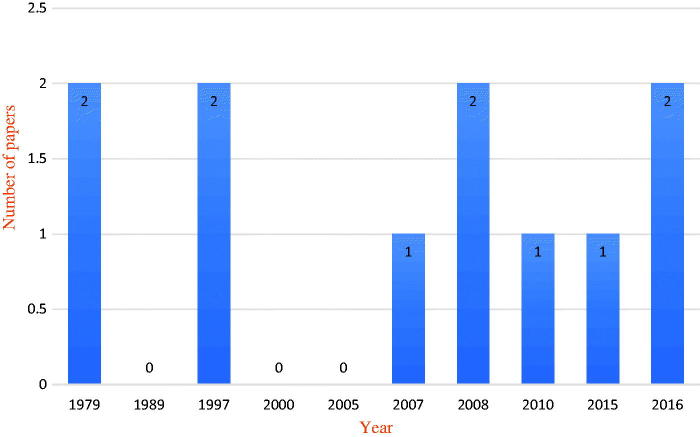
Classification of research papers by year of publication.

The following main characteristics of the reviewed studies were extracted from each study: publications, year, objectives, study details, methodology, results and conclusions (see Table 1). As shown in Table 1, only two of the nine studies (36.36%) included in this review were published by the end of 2000. Of the remaining seven studies, four were published by the end of 2010 (36.36%). Finally, three studies (27.27%) were published between the beginning of 2015 and the end of 2016 (Figure 2).

On the one hand, renal image studies are clustered as follows: Glomeruli Segmentation and analysis of the Glomerular basement membrane (55/55%), Blood vessels and tubules classification and detection (22/22%), and The Grading of renal cell carcinomas (22/22%).

**Figure 3.**
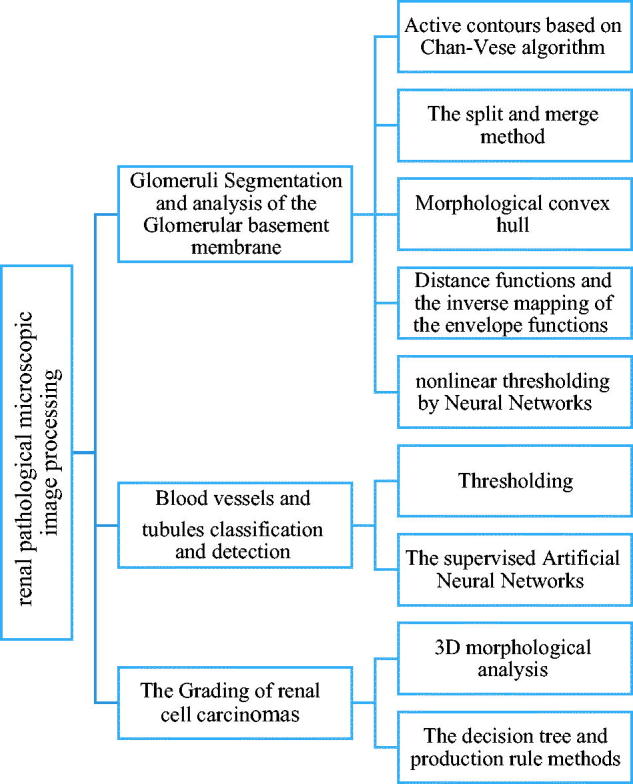
Mind map of the renal image processing techniques.

In the next sections, we briefly describe the principles of these studies; methods are presented including a conceptual mind-map as depicted in Figure 3, closing with the observed beneficial and challenging effects as a result of a fusion between the clinical and the computer science perspective.

### 3.1. Glomeruli segmentation and analysis of the glomerular basement membrane

#### 3.1.1. Active contours based on Chan-Vese algorithm

The ‘active contours’ begin by an initialized contour and actively deform themselves to the desired border at the same time reducing the defined energy in each iteration until it converge [18]. Convergence is achieved when reaching a balance between the External powers that attracts the contour to its place and the ‘Internal’ powers which keeps it smooth, usually by maintaining some function of its curvature. Chan-Vese algorithm depends on global properties such as region areas and intensities, instead of account local properties, such as gradients. Let Ω be bounded open set of Ɽ2, with ∂Ω as its boundary. Let $\mu_{0}:\Omega \to$Ɽ be a given image, and, C(s): [0, 1]→Ɽ^2^ parameterized curve. Let us denote the region inside C as ω and outside C as Ω′\ω. Let, $c_{1}$ denotes the average intensity of pixels inside C, and $c_{2}$ indicates the average intensity of pixels outside C. Minimizing the energy function is objective of the Chan-Vese algorithm $F\left(c_{1},c_{2},C\right)$, defined by:

(1) $$F\left(c_{1},c_{2},C\right)=\mu .\mathrm{Length}(C)+v.\mathrm{Area}\left(\mathrm{inside}\left(C\right)\right)+\lambda_{1}{\int \left|\mu_{0}\left(x,y\right)-c_{1}\right|}^{2}d\mathrm{xdy}+\lambda_{2}{\int \left|\mu_{0}\left(x,y\right)-c_{2}\right|}^{2}d\mathrm{xdy}$$

**Figure 4.**
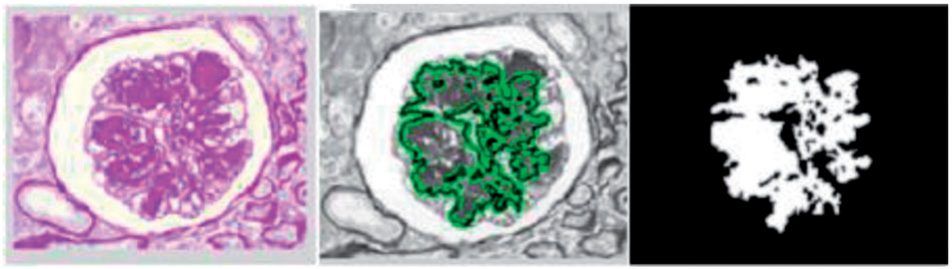
Counter based segmentation of glomerular images.

Both inside and outside *C*, μ*≥* 0*, v ≥* 0*, λ*_1_, and *λ_2_≥* 0 are the parameters which are defined by the user (Figure 4).

#### 3.1.2. The split and merge method

**Figure 5.**
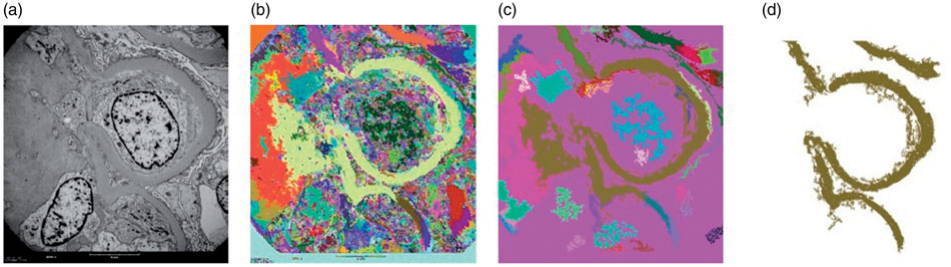
(a) A TEM image of a renal biopsy sample; image size 485 *×* 512 pixels. (b) The result of application of the S&M algorithm to the image in (a). (c) Result of filtering by size and by shape applied to the image in (b). Size threshold equal to 0.03. Compactness threshold equal to 0.9. (d) The result in (c) after marking the regions corresponding to the GBM and removal of artifacts [12].

The SAM method [19] is one of the region-based segmentation methods. This approach specifies the region boundaries using discontinuities of intensity levels [20]. First input image is considered as a primary region then the region is divided into quadregions. A homogeneity criterion is checked for each quadregion. If this criterion is not satisfied, this quadregion is splitted to more quadregions. When all the regions satisfy the homogeneity criterion, splitting is stopped. If the homogeneity criterions of neighboring regions are close together, they can be merged. Regions based on size and shapes are rejected, if required, manually add GBM segments and remove artifacts [12,19] (Figure 5).

#### 3.1.3. Distance functions and the inverse mapping of the envelope functions

**Figure 6.**
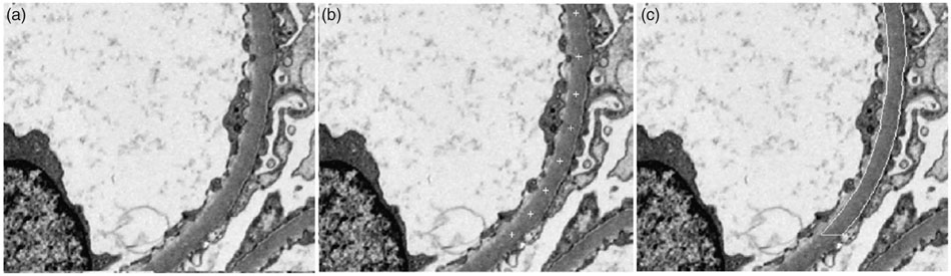
(a) Original image acquired at magnification of 2k, (b) user specified sequence shown by the cross signs along the segment of BM to be measured, (c) two segmented sub-BM segments indicated by the shapes [14].

An electron microscopic image can be represented by a 2D discrete sequence *x* = {*x* (*n*1, *n*2)} whose elements are valued between 0 and 255 and defined in a rectangular domain of *S* = *{*(*n*1, *n*2) |0≤*n*1 <*N*1; 0≤*n*2 <*N*2*}*. A sequence of center points along the center of the GBM are manually specified. A vector sequence **c** = {**c** (*k*) |0≤*k* <* K*}, where **c**(*k*) = (*c*1(*k*),*c*2(*k*)) is provided, and *K* is the total number of center points manually inputted. The vectors are sparsely located along a segment of the GBM. This algorithm requires only minimum user inputs whose positions are much less critical and determines the GBM thickness based on the local contents in the images. In the case of a narrow angle range, an appropriate mapping of the local image into a smaller rectangular image converts the usually curved GBM segment into a straight one. Two distance functions of the non-GBM pixels to the straight central line on the two sides are evaluated and their smooth envelope functions are derived. The inverse mappings of the two envelopes produce the boundaries of the GBM segment. The average GBM thickness is estimated as the ratio of the area inside the recognized GBM segment to the length of the central line. Long GBM segments with wide-angled vector sequence of manual inputs are partitioned into multiple narrow-angled sub-segments prior to the segmentation procedures [14] (Figure 6).

#### 3.1.4. Nonlinear thresholding by neural networks

**Figure 7.**
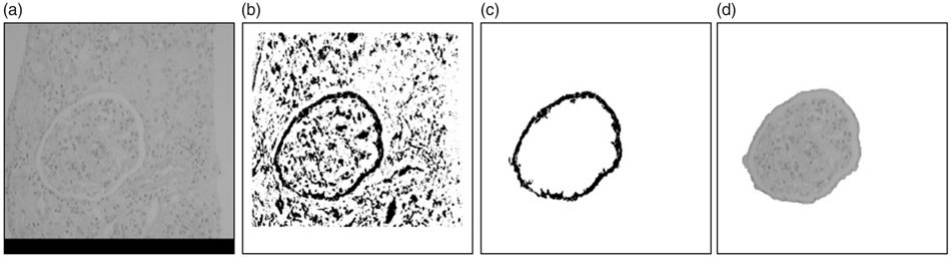
(a) Original image, (b) enhancement, (c) boundary extraction, (d) glomerulus.

The typical three-layer BP neural network is used to construct the thresholding surface. The input is a column vector consisting of 84 different values given by feature operator, namely, the node number of input layer is 84. The node number of middle layer is 100. The node number of output is 1, denoting the object (black points) or the background (white points). One image is chosen randomly from the tissue sections as teacher image. 100 pixels are chosen manually and respectively from the cavum boundary and the background as teacher signals. Here, the patterns of teacher signals have been remembered in the network. If the characteristics reflecting the two patterns is disjunctive (even nonlinear disjunctive), the value of convergence error will be zero. In this method, the characteristic of this can be described as the following equation.

(2) $$\{\begin{array}{ccc} f(i,j)\in \left\{\text{Boundary}\right\} & {\nabla f(i,j)|}_{S_{\mathrm{in}}}\in \Omega_{1} & {\nabla f(i,j)|}_{S_{\mathrm{out}}}\in \Omega_{2}\\ f(i,j)\in \left\{\text{GlassRegion}\right\} & {\nabla f(i,j)|}_{S_{\mathrm{in}}}\in \Omega_{1} & {\nabla f(i,j)|}_{S_{\mathrm{out}}}\notin \Omega_{2}\\ f(i,j)\in \left\{\text{Others}\right\} & {\nabla f(i,j)|}_{S_{\mathrm{in}}}\notin \Omega_{1} & {\nabla f(i,j)|}_{S_{\mathrm{out}}}\in \Omega_{2} \end{array}$$

where, Ω_1_ is the pixel set of the small difference values in $S_{\mathrm{in}}$, Ω_2_ is the pixel set of the irregular difference values in $S_{\mathrm{out}}$, f(i,j) is pixel. It is difficult to determine the threshold or find an equation to calculate the threshold based on this feature operator. So the neural network is used to construct a nonlinear thresholding surface to enhance the cavum boundary [13] (Figure 7).

#### 3.1.5. Morphological convex hull

A set A is said to be convex if the straight line segment joining any two points in A lies entirely within A. The convex hull Hof an arbitrary set Sis called the convex deficiency of S. There is a simple morphological algorithm for obtaining the convex hull, C(A) of a set A. If $B^{i}$, i=1,2,3,4, represent the four structuring elements. The procedure consists of implementing the equation:

(3) $$X_{k}^{i}=(X_{k-1}\;B^{i})\cup X_{k-1}^{i}\;i=1,2,3,4\;\text{and}\;k=1,2,3,..$$

where $X_{0}^{i}=A$. W is a small window surrounds $B_{{}_{2}}^{i}=W-B_{{}_{1}}^{i}$. When the procedure converges (i.e., when $X_{k}^{i}=X_{k-1}^{i}$), $D^{i}=X_{k}^{i}$. Then, the convex hull of A is

(4) $$C\left(A\right)=\overset{4}{\underset{i=1}{\cup }}D^{i}$$

**Figure 8.**
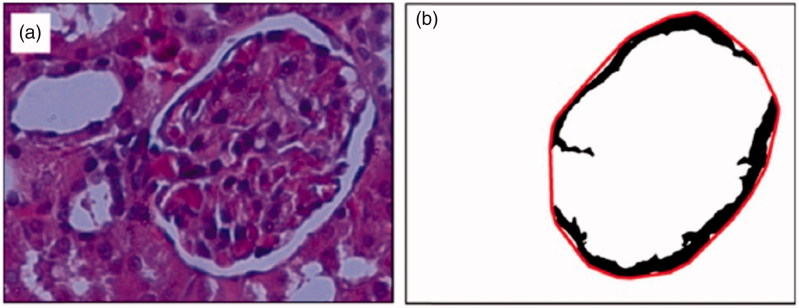
Glomerulus detection algorithm for diameter measurement: (a) original RGB image (b) convex hull.

The regular boundary areas of cytoplasm and polar body are achieved using the convex hull algorithms [19]. Result of this method is shown in Figure 8.

### 3.2. Blood vessels and tubules classification and detection

#### 3.2.1. Thresholding

**Figure 9.**
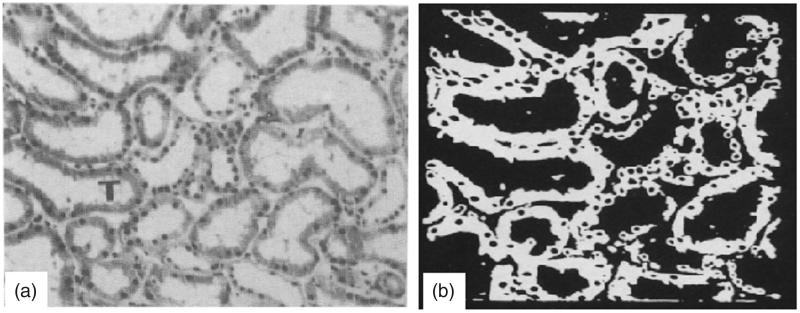
The histological image of the normal human kidney. (a) The tubule to be reproduced in the following pictures are located around space marked by a ‘T’. (b) Thresholded image.

Thresholding is an essential region-based image segmentation technique that is particularly useful for scenes containing solid objects resting on a contrasting background. It is computationally simple and never fails to define disjoint regions with closed, connected boundaries. The operation is used to distinguish between the objects of interest (also known as the foreground) and the background on which they lay. The output is either the label ‘object’’ or ‘background,’ which can be represented as a Boolean variable. In general, a gray-level thresholding operation can be described as:

(5) $$G(x,\;y)=\{\begin{array}{cc} F & \mathrm{if}\;I(x,y)\geq T\\ B & \mathrm{if}\;I(x,y)<T \end{array}$$

where I(x, y) is the original image, T is the threshold, G(x, y) is the threshold image, and F corresponds to the foreground labeled with either a designated gray-level value or the original gray level, I(x, y). Thus, all pixels at or above the threshold are assigned to the foreground and all pixels below the threshold are assigned to the background, which is labeled B [19]. Figure 9 shows the histological threshold image. The image section to be reproduced in thresholded form is centered around the tubules marked by a T. Each tubule consists of a white lumen surrounded by a gray epithelial lining in which the nuclei appears as dark spots.

#### 3.2.2. The supervised artificial neural networks

**Figure 10.**
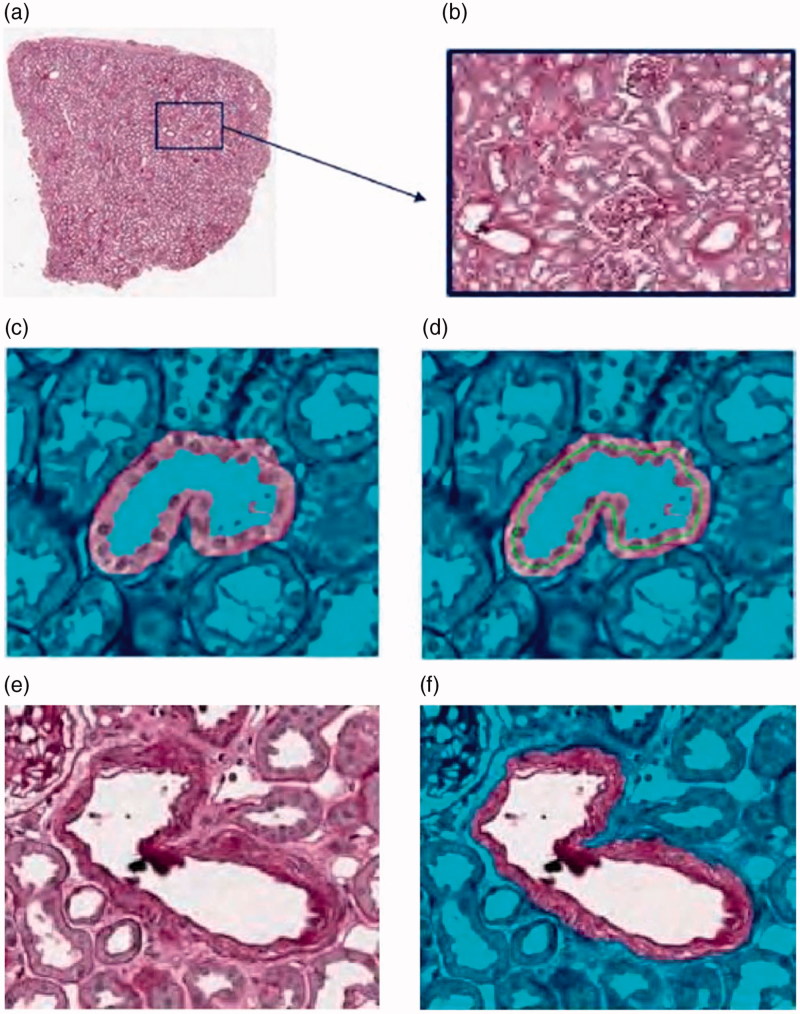
(a) Original RGB image, (b) Sub-ROI of RGB image (a), (c) Tubule, (d) Tubules centerline, (e) ROI vessel, (f) vessel membrane.

An Artificial Neural Network (ANN) is an information processing paradigm that is inspired by the way in which the biological nervous system processes information: ANN have been extensively used to model complex input/output relations for diverse aims, such as classification, control, optimization, estimation in numerous applications fields, such as medical, robotic, manufacturing, transportation, financial, and many more [21,22,23]. The key element of the ANN paradigm is the structure of the information processing system, which is composed of a large number of highly interconnected processing elements (neurons) that cooperate to solve specific problems. All connections among neurons are characterized by numerical values (weights) that are updated during the training phase. The computation performed by the ith neuron can be expressed as a nonlinear function of the weighted sum of the neuron outputs connected to the ith neuron. The ANN is trained by a supervised learning process: in the training phase the network processes all the input/output pairs presented by the user, learning how to associate a particular input to a specific output and trying to extend the acquired information also to cases that do not belong to the training set spectrum. Typically, the ANN input dataset is subject to preprocessing. One such method is the normalization of variables so as to have a uniform distribution, with data that are normalized in the [0, 1] range (Figure 10).

### 3.3. The grading of renal cell carcinomas

#### 3.3.1. 3D Morphological analysis

Connected-component labeling is a preprocessing step and is typically performed before the quantitative analysis. In 3D morphological analysis, a new 3D labeling method based on slice information are used [24]. After applying a 2D connected-component labeling algorithm to each slice by using a contour-tracing technique [25], the labeled information to detect objects in the next slice that might belong to the same object are applied. For example, if there was an object common to the kth and (k+1)th image slices, the center pixels of each object is compared to determine if the objects were connected. If they were connected, the same label to both is assigned. If a common object existed in consecutive slices but the center pixels were unconnected owing to holes in each object, the remaining regions of the object are compared, omitting the center pixels, to determine whether they were connected. If the two objects were connected, they are assigned the same label; if the two objects were not connected, they are assigned a different label to the objects in the(k+1)th image. The following parameters define the characteristics of a cell nucleus in three dimensions [26,27].

##### 3.3.1.1. Volume

This is determined by the total number of voxels in the nucleus. The number of voxels multiplied by the size of a voxel gives the size of a cell nucleus in standard units.

##### 3.3.1.2. Surface area

The area of a 3D cell nucleus can be approximated by the number of voxels belonging to the nucleus that have at least one neighboring background voxel. However, to find all the surface voxels, the relationships between voxels must be recomputed using the connectivity operation. Heron’s formula as an alternate way to measure surface area are used, calculating the area of a triangle directly in terms of the lengths of the three sides, since the rendered surfaces consist of triangles in computer graphics. After calculating each of the triangle areas, we obtained the total surface area using the sum of all triangle areas.

(6) $$S=\;\sqrt{s(s-a)(s-b)(s-c)}$$

where *a, b, c* are the lengths of the sides of a triangle and

(7) s=(a+b+c)/2.

##### 3.3.1.3. Spherical shape factor

**Figure 11.**
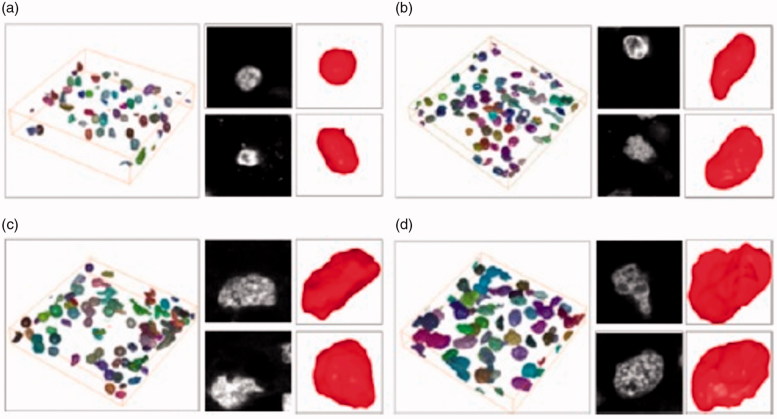
Results of the three-dimensional visualization: surface-rendering image for cell nuclei of (a) Grade 1, (b) Grade 2, (c) Grade 3, and (d) Grade 4 [11].

This represents how similar the shape of the cell nucleus is to a sphere. If *A* is the surface area of the nucleus and *V* is the volume of the nucleus, then the spherical shape factor is defined as $36\pi v^{2}/A^{3}$ (Figure 11) [11].

#### 3.3.2. Decision tree and production rule system from the C4.5 algorithm

**Figure 12.**
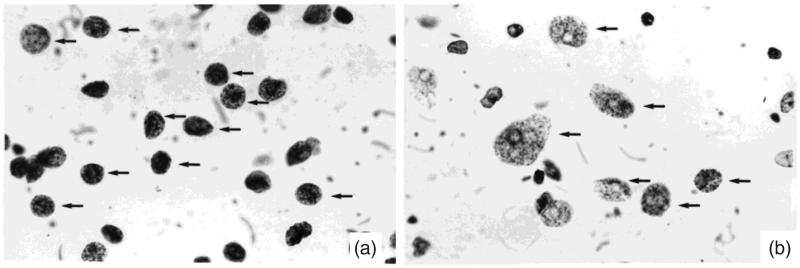
Cytological aspect (Feulgen staining; #1000) of (a) a low-grade and (b) a high-grade renal cell carcinoma [10].

These models train on data for classification and regression problems based on decisions fork in tree structures. The C4.5 algorithm uses a measure of information gain ratio for selecting an input variable in each node. This variable selection process is based on the precise probabilities calculated from the training set [28] (Figure 12).

##### 3.3.2.1. Production rule system (PR)

A PR algorithm directly generates logical classification rules with the form: ‘IF (the test on feature *Xi* is satisfied) and (the test on feature *X_j_* is satisfied) and (the test on feature *X_k_* is satisfied), THEN the class will be *Cx*’. In C4.5, a rule-generating mechanism is also available. Its purpose is to generate a production rule classifier that is usually about as accurate as a pruned true and more easily understood. The final rules are arranged according to an order of priority and the first rule that covers a case is taken as the operative one. A default class is imposed on all cases not covered by any of the final rules, this being the class most frequently observed among these cases [29].

### 3.4. Beneficial and challenging effects

The challenge for the development of Glomeruli Segmentation and analysis of the Glomerular basement membrane is observed [12–15,17]. In the last decade, only one method for the classification of blood vessels and tubes is presented [16]. Despite the importance of renal cell carcinomas grading, much research has not been done in recent years [10,11]. Pathologic images provide very accurate information from the tissue. Also, there are some very important issues regarding the examination of kidney diseases with image analysis tools that hope to be considered in future research.

## 4. Discussion and conclusions

The study of pathological microscopic image processing methods for renal diagnosis can provide significant insight into the conceptual basis of this methods growing domain. The current systematic review study is on articles written during 1979–14 June 2017. In related studies selecting process, databases were searched by using renal, pathological microscopic image, and image processing methods terms. Article selection criteria are considered in this process. In the first stage, their titles and abstracts was investigated at last, the articles were selected based on full text reading. The findings of this article show that renal pathological image processing techniques key issues are classified into three important categories of methods, such as glomeruli segmentation, blood vessels and tubules classification, and the grading of renal cell carcinomas.

The selection of the threshold value is crucial to the success of a thresholding operation in tubules detection. Unless the object in the image has very steep sides, any variation in threshold value can significantly affect the boundary position and thus the overall size of the extracted object [19]. Also, Neural Networks methodology is limited in terms of knowledge visualization. Additionally, determining the adequate size of the hidden layer is vulnerable to poor approximations (caused by lack of nodes) and over fitting (from excessive nodes) [30].

However, active contours are effective in medical image segmentation but they have several drawbacks as follows: They are sensitive to local minima states, which can be counteracted by simulated annealing techniques. Minute features are often ignored during energy minimization over the entire contour. Their accuracy depends on the convergence policy [31].

The purposed algorithm given in reference [14] requires user inputs whose positions are necessary for the determination of GBM thickness. Moore neighborhood contour tracking method can be used for glomeruli segmentation in further works. Moreover, Image processing in 3D space has a lot of computational complexity, so by using powerful texture analysis techniques such as the local binary pattern, we can find the appropriate results in terms of renal cell carcinomas grading from two-dimensional images.

In recent articles, glomeruli segmentation and analysis of the glomerular basement membrane have become more important (Figure 3). Blood vessels and tubules classification, detection, and The Grading of renal cell carcinomas can be studied more detail in further research.

Only English-language publications are considered in this review, while different language articles can be discussed in further works. Also, no *special renal disease was persued in this review.*
